# HFD and HFD-provoked hepatic hypoxia act as reciprocal causation for NAFLD via HIF-independent signaling

**DOI:** 10.1186/s12876-020-01515-5

**Published:** 2020-11-03

**Authors:** Xiaofang Zhang, Caoxin Huang, Xuejun Li, Zhaoshui Shangguan, Wenjing Wei, Suhuan Liu, Shuyu Yang, Yijie Liu

**Affiliations:** 1 Xiamen Diabetes Institute, The First Affiliated Hospital of Xiamen University, 55 Zhenhai Road, Xiamen, 361003 China; 2 Xiamen Clinical Medical Center for Endocrine and Metabolic Diseases, Xiamen, China; 3 Fujian Province Key Laboratory of Diabetes Translational Medicine, Xiamen, China; 4 Department of Endocrinology and Diabetes, The First Affiliated Hospital of Xiamen University, Xiamen, China; 5 Research Center for Translational Medicine, The First Affiliated Hospital of Xiamen University, Xiamen, China; 6 Siming Branch, The First Affiliated Hospital of Xiamen University, 55 Zhenhai Road, Xiamen, 361003 China

**Keywords:** NAFLD, Hypoxia, HIF, Steatosis

## Abstract

**Background:**

The occurrence of non-alcoholic fatty liver disease (NAFLD) is found to be higher in patients with obstructive sleep apnea (OSA), which is characterized by intermittent hypoxia. Activation of hypoxia-inducible factors has been shown in the development and progression of NAFLD, implying a cause and effects relationship between NAFLD and hypoxia. The present study was designed to investigate the interaction of lipotoxicity and hypoxia in the pathogenesis of NAFLD using mice model with high-fat diet (HFD) feeding or hypoxic treatment.

**Methods:**

NAFLD model was induced in mice by HFD feeding, and in cultured primary hepatocytes by administration of palmitate acid. Mouse hypoxic model was produced by placing the mice in a Animal incubator with oxygen concentration at 75% followed by a 21% oxygen supplement. Hypoxic condition was mimicked by treating the hepatocytes with cobalt chloride (CoCl_2_) or 1% oxygen supply. Pimonidazole assay was conducted to evaluate hypoxia. Lipid metabolic genes were measured by real-time polymerase-chain reaction. HIF-1α and HIF-2α genes were silenced by siRNA.

**Results:**

HFD feeding and palmitate acid treatment provoked severe hepatic hypoxia along with TG accumulation in mice and in cultured primary hepatocytes respectively. Conversely, hypoxia induced hepatic TG accumulation in mice and in cultured primary hepatocytes. Hypoxic treatment inhibited the expression of lipolytic genes, while increased the expression of lipogenicgenes in mice. Although both lipotoxicity and hypoxia could activate hepatic hypoxia-induced factor 1α and 2α, while neither lipotoxicity- nor hypoxia- induced hepatic steatosis was affected when HIF was knocked down.

**Conclusions:**

HFD resulted in hepatic TG accumulation and concomitant hypoxia. Conversely, hypoxia induced hepatic TG accumulation in mice and in cultured heptocytes. Thus lipotoxicity and hypoxia might work as reciprocal causation and orchestrate to promote the development of NAFLD.

## Background

Non-alcoholic fatty liver disease (NAFLD) is increasingly recognized as one of the most common liver disease, which frequently progresses into nonalcoholic steatohepatitis (NASH), cirrhosis and hepatocellular carcinoma [1, 2]. Due to the lack of efficient treatment except life style intervention, NAFLD is associated with high morbidity rate affecting more than 30% of the adults and 10% of children in western countries, or even higher in patients with diabetes and/or obesity [3].

Multiple factors, including environmental and genetic factor, contribute to the development of NAFLD, making the pathogenesis of NAFLD complex thus hard to be fully elucidated [4]. Although the initial “two hit” hypothesis was generally accepted, emerging evidence promptmulti-hit process involving a variety of overlapping pathways, including lipotoxicity, oxidative stress, gut dysbiosis et al. [5]. Recently, a growing number of studies have linked obstructive sleep apnea syndrome (OSAS) with NAFLD, by showing that OSAS patients predisposes to the development of NAFLD from pediatrics to the aged, and the mainly relevant is the burden of nocturnal hypoxia independently of BMI and diabetic status [6]. OSAS, characterized by partial or total obstruction of the upper respiratory tract in sleep, represents a situation of intermittent hypoxia [7]. In addition, blood TG levels raised in a young group of newcomers to altitude after being exposed chronically for 8 months [8], and two-fold increase of hepatic triglycerides was observed in the chronic hypoxia rats [9].

Indeed, activation of hypoxia-inducible factor (HIF) has been showed to induce hepatic lipid accumulation and NAFLD progression [10, 11]. In addition, chronic intermittent hypoxia (CIH)- treated obese mice, but not lean mice, developed insulin resistance and NAFLD, suggesting that obese situation might predispose the mice to hypoxia-related hepatic injuries [12, 13]. While the interaction of lipid accumulation and hypoxia in the pathogenesis of NAFLD, and the reason why obese subjects are more predisposed to NAFLD still needs further clarification. The present study was designed to investigate the individual- and interactional role of high-fat diet (HFD) and hypoxia in the development of NAFLD.

## Methods

### Cell culture

Primary hepatocytes were dissociated from anesthetized adult mice by a non-recirculating collagenase perfusion through theportal vein. The isolated cells were then filtered through a 100 μm pore size mesh nylon filter, and 5*10^5 cells grown in 3.5 cm petri-dishes cultured with hepatocyte medium (HM) (ScienCell, UT, USA), supplied with 5% fetal bovine serum (FBS), 1% Penicillin–Streptomycin (P/S), in a 37 °C incubator (Sanyo, Guangzhou, China) containing 5% carbon dioxide.

### Animals

C57BL/6N male mice aged 8 weeks were purchased from Shanghai SLAC Laboratory Animal Co. Ltd. (Shanghai, China) separated randomly into groups of three to five mice per cage. All procedures were approved by the Committee for Animal Research at Xiamen University, and were performed according to the guidelines for animal care and use. Mice were raised under specific-pathogen free conditions in a 12-h light (7:00–19:00)/12-h dark (19:00–7:00) cycle with free access to water and normal mouse chow. Room temperature was stably maintained at 22 ± 1–2 °C. To induce NAFLD, mice were fed with high-fat-diet (HFD) contained 60% kcal fat for 8 weeks. At the end of week 8, body weight was monitored and mice were sacrificed after 10–12 h of fasting. Hypoxia mice model were produced by housing the mice in 75% oxygen supplement cage for 5 days started on postnatal days 7 (P7), and then placing in 21% oxygen environment for 5 days. Mice were sacrificed by cervical dislocationon postnatal days 17 (P17).

### Quantitative real-time RT-PCR

Total RNA from the liver was extracted using total RNA extraction kit (TIANGEN, DP419). Synthesis of cDNA was conducted using a FastKing RT Kit (TIANGEN, KR116). Quantitative real-time PCR (qPCR) was performed using SYBR Green I Master( Roche, 04887352001) in an LC480 thermal cycler (LightCycler 480). The mRNA value of each gene was normalized to that of β-Actin.

### Histological staining

For hematoxylin and eosin staining (H&E staining), liver tissues were fixed in 10% neutral buffered formalin and embedded in paraffin. Sections were subjected to standard H&E staining. For oil red O staining, liver tissues were fixed in 4% paraformaldehyde in PBS, embedded in optimum cutting temperature compound (OCT), stored at − 20 °C, and cryosectioned at use. Frozen liver sections were stained with 0.5% oil red O according to standard procedures.

For hypoxia assay, hepatocytes were treated with 100 mM concentration of pimonidazole for 2 h, fully permeabilized with PBS containing 0.2% Triton, blocked with 3% BSA and incubated with Hypoxyprobe-1 which were labeled with fluorescein isothiocyanate (FITC) (1:100), the nuclei were stained with DAPI (VECTASHIELD with DAPI, H-1200). Pictures were taken using an Olympus BX-51 microscope.

### Measurement of TG content

Mouse livers were weighed, and were then added 9 times volume of 0.1 M concentration of PBS. Liver samples were mechanically homogenated in ice bath, and were then centrifuged at 2500 rpm/ min for 10 min. The supernatant was collected, and were incubated with working solution at 37 °C for 10 min following the manufacture’s instruction, and were then read with imark microplate absorbance reader at 510 nm wave length (Nanjing jiancheng Bioengineering Institute, A110-1).

### Western Blotting

Hepatocytes and liver were lyzed with RIPA lysis buffer (Millpore, MA, USA). The concentration of total protein in the supernatant was measured by BCA kit (Thermo Fisher Scientific, MA, USA). The primary antibody HIF-1α (Novus Biologicals, 1:1000), HIF-2α (Novus Biologicals, 1: 1000), α-Tubulin (Cell signaling technology, 1: 1000), β-actin (Cell signaling technology, 1:1000), FITC-MAb1(Hypoxyprobe, Inc,1:10,000) were incubated in 4 °C overnight. The secondary antibodies (goat anti Rabbit, 1:5000, goat anti mus, 1:10,000, rabbit anti-FITC HRP1:10,000) were incubated 1 h in room temperature. ECL luminescence liquid (Lulong, Xiamen, China) was used while the light emitted was captured by the machine followed by the pictures being saved.

### siRNA transfection

To perform small-interfering RNA (siRNA) transfection, cultured primary hepatocytes were treated with HM and incubated for 24 h. siRNA targeting HIF-1α (no. CAAGCAACUGUCAUAUAUAdTdT-UAUAUAUGACAGUUGCUUGdTdT; Sigma-Aldrich) and HIF-2α (no. GACUUACUCAGGUAGAACUdTdT-AGUUCUACCUGAGUAAGUCdTdT; Sigma-Aldrich), or a scrambled control (UUCUCCGAACGUGUCACGUTT-ACGUGACACGUUCGGAGAATT; Sigma-Aldrich) was transfected using Lipofectamine™ 2000 Transfection Reagent (no. 11668019; Invitrogen) according to the manufacturer’s instructions, change fresh HM after 6 h’ transfection. Drug was added to the medium 24 h after transfection.

### Statistical analysis

Data were prsented as means ± S.E.M, and were analyzed by one-way ANOVA using Graphpad prism 5.0 software. Difference was considered statistically significant when P < 0.05.

## Results

### HFD feeding induced hepatic steatosis and provoked hepatic hypoxia in mice

**Fig. 1:**
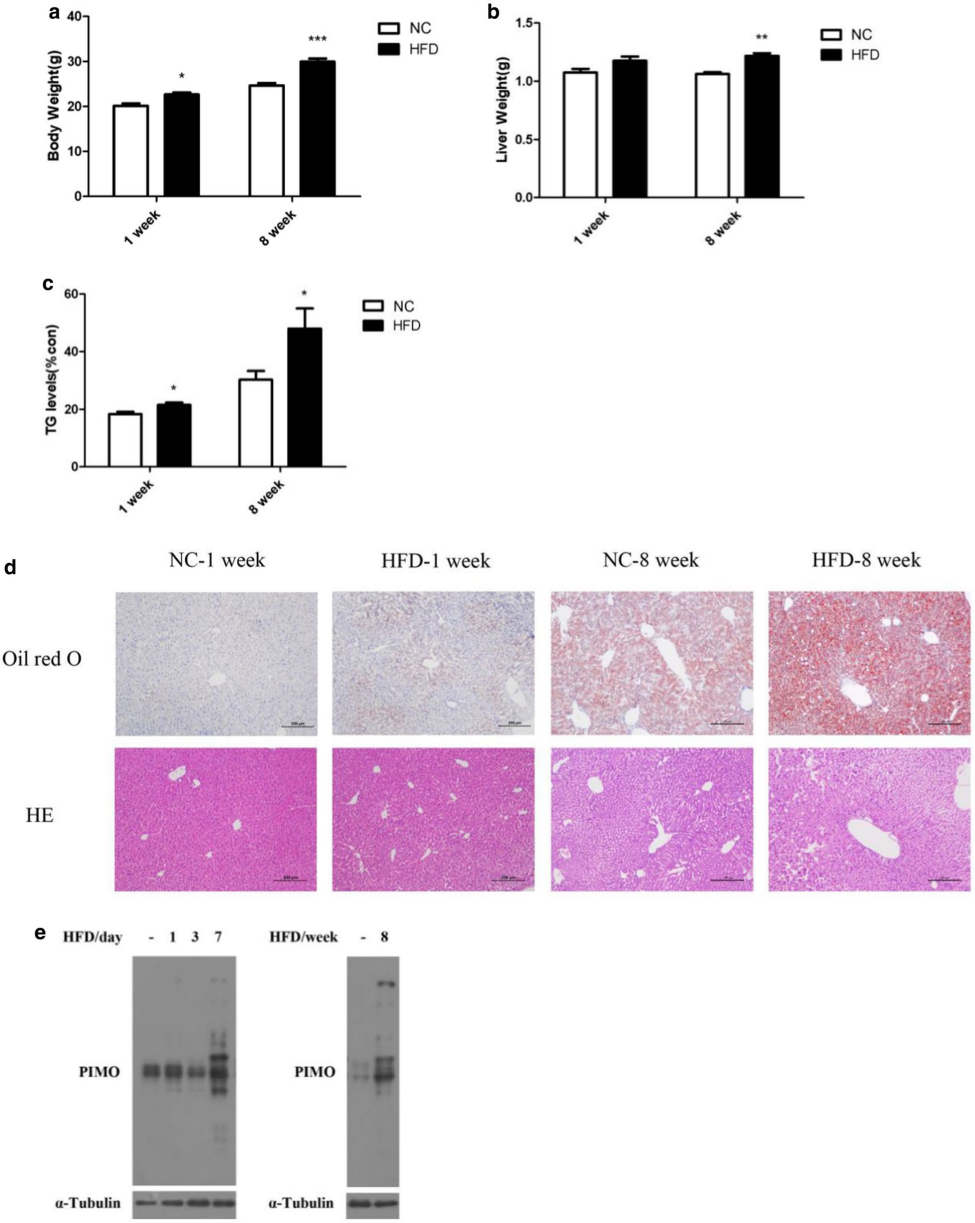
The effect of HFD on fat accumulation and hypoxia conditions in the liver. **a** Body weight; **b** liver weight; **c** TG levels; **d** oil red O and HE staining, (original magnifications, × 100); and **e** expression of hypoxic proteins. Data are shown as the mean ± SEM, n = 5–9 in each group. *P < 0.05; **P < 0.01; ***P < 0.005

Mice were fed on HFD for 7 days, representing an acute effect, and 8 weeks, representing a chronic effect respectively. We found that both acute and chronic HFD treatment increased fat accumulation predominantly started from pericentral region of the liver acinus, showed by increased body weight, liver weight and hepatic TG content (P < 0.05) (Fig. 1a–d). We observed a robust accumulation of hypoxic proteins, which appeared on the seventh day after HFD feeding and lasted for 8 weeks, indicating a hypoxia injury was induced by HFD concomitantly with extra fat deposit (Fig. 1e).

### Palmitic acid induced lipid accumulation and concomitantly provoked hypoxia in cultured primary hepatocytes

**Fig. 2:**
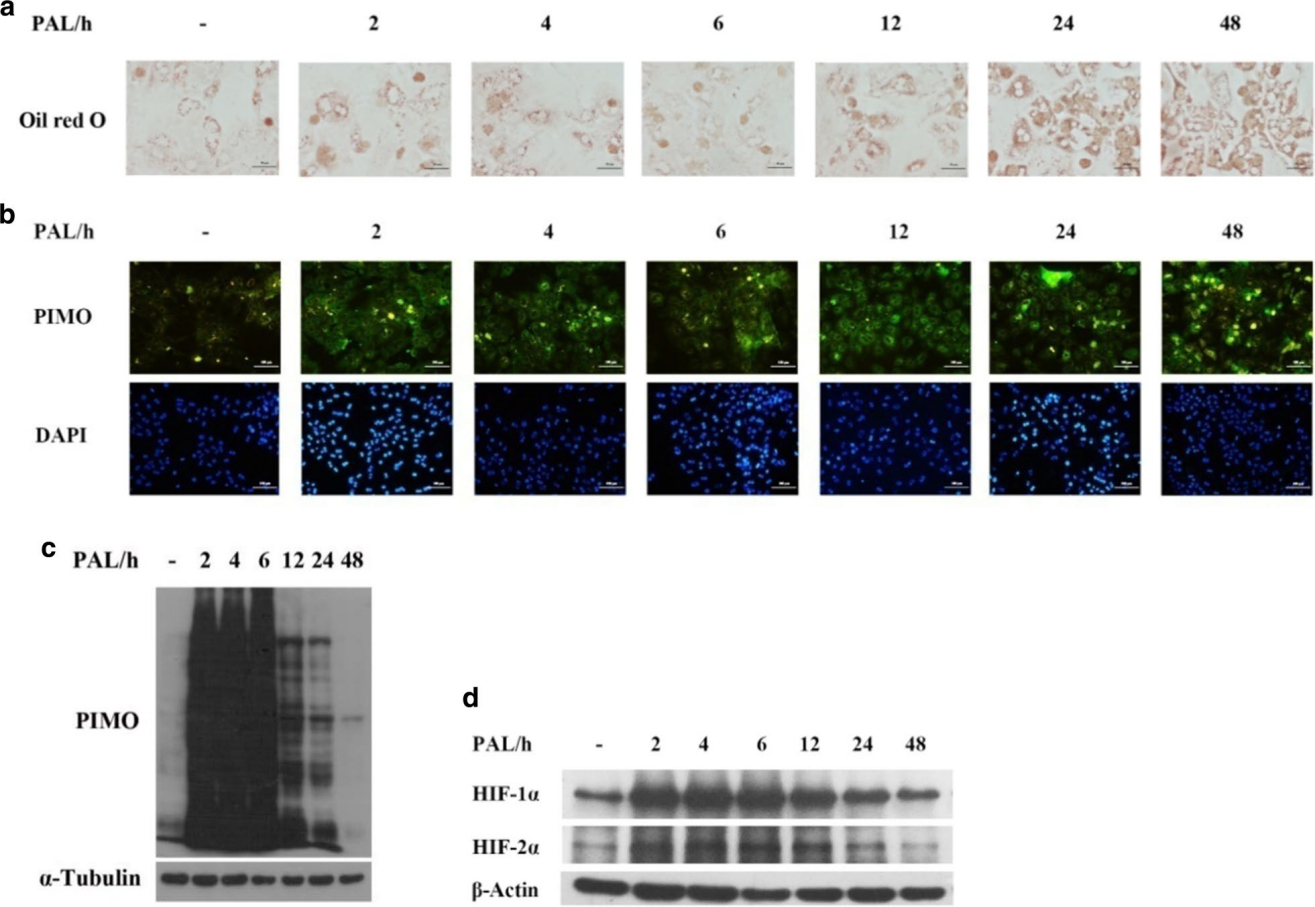
The effect of palmitate acid on fat accumulation and hypoxia conditions in cultured primary hepatocytes. **a** Oil red O staining; **b** expression of hypoxic proteins detected by immunofluoroscence staining; **c** expression of hypoxic proteins detected by Western Blotting; **d** expression of HIF-1α and HIF-2α. Data are shown as the mean ± SEM, n = 5

Primary hepatocytes were treated with 0.4 mM palmitic acid for 2, 4, 6, 12, 24 and 48 h. As expected, we found that palmitic acid treatment increased heptatic lipid accumulation in a time-dependent manner (Fig. 2a). At the same time, we observed a robust accumulation of hypoxic proteins in palmitic acid treated hepatocytes (Fig. 2b, c). Consistent with the upregulation of hypoxic proteins, the expression of HIF-1α and HIF-2α were increased simultaneously (Fig. 2d).

### Hypoxia- induced hepatic steatosis is independent of HIF activation in cultured primary hepatocytes

**Fig. 3:**
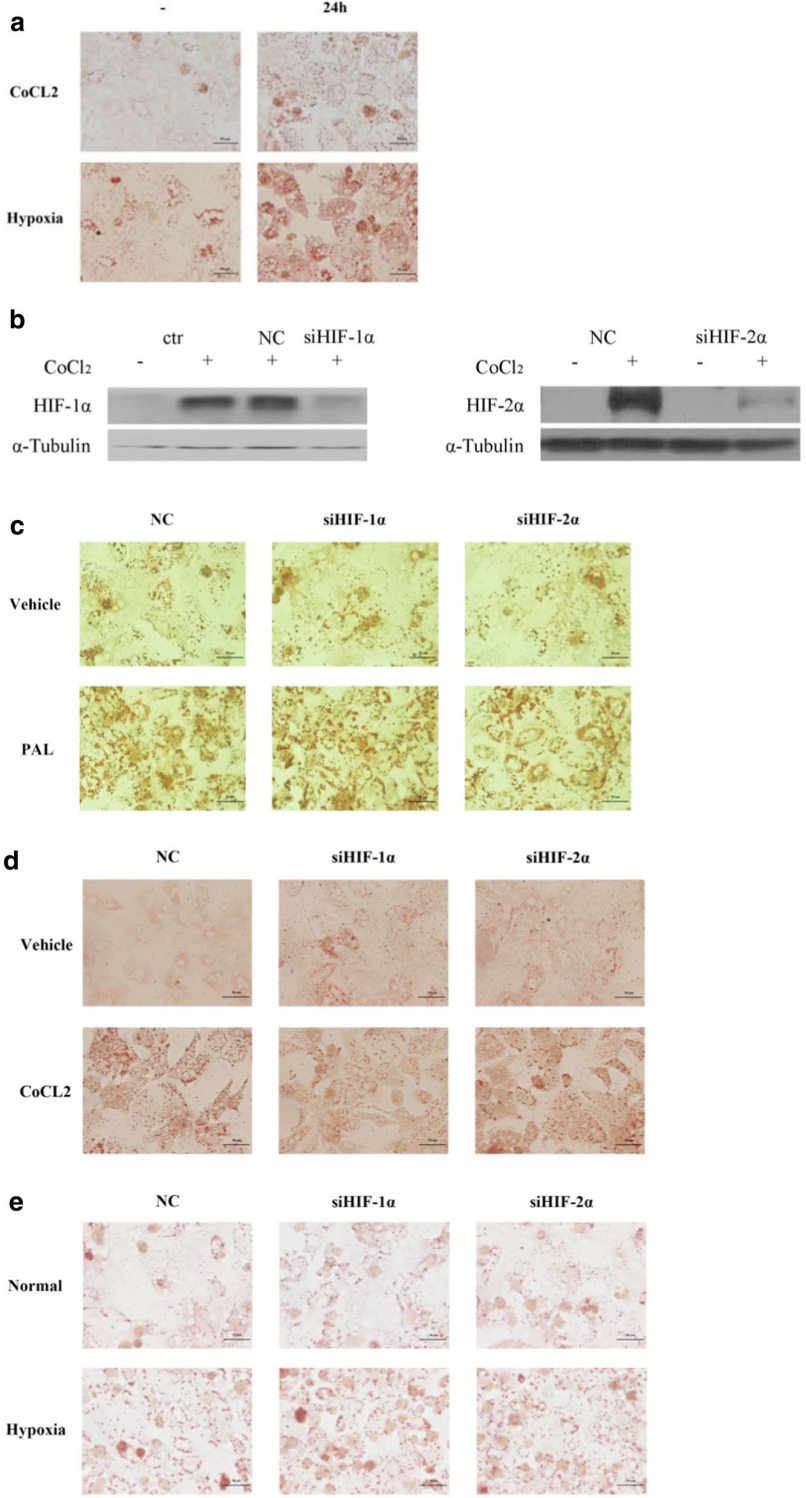
The effect of FFA- or hypoxia-induced TG accumulation in primary hepatocytes were not HIFα dependent. **a** Oil red O staining. **b** HIF1- and 2α knockdown in primary hepatocytes by siRNA. Oil red O staining in FFA- (**c**), CoCl_2_- (**d**) and hypoxia-treated. **e** Primary hepatocytes with HIF-1α and HIF-2α deletion. Data are shown as the mean ± SEM, n = 5

We found that the quantity of lipid droplets within the primary hepatocytes increased dramatically when the cells were cultured under hypoxic conditions, with 0.4 mM Cobalt chloride (CoCl_2_) treatment or cultured in a 1% O_2_ environment (Fig. 3a). HIF has been showed to play a pivotal role in regulating hepatic lipid metabolism, and in the pathogenesis of NAFLD [14]. Cobalt chloride is frequently used as a hypoxic inducer mimicking HIF effects. We observed that CoCl_2_ could activate both HIF-1αand HIF-2α, which was abolished when the cells were treated with small molecule interference RNA (siRNA) for HIF-1α and HIF-2α respectively (Fig. 3b). Neither palmitic acid—nor hypoxia- induced hepatic steatosis was ameliorated when HIF signaling was blocked by siHIF-1αand siHIF-2α respectively (Fig. 3c–e), suggesting that HIF did not play a causal role here mediating palmitic acid- and hypoxia- induced hepatic steatosis, although it was significantly activated.

### Hypoxic treatment resulted in hepatic hypoxia and steatosis in mice

**Fig. 4:**
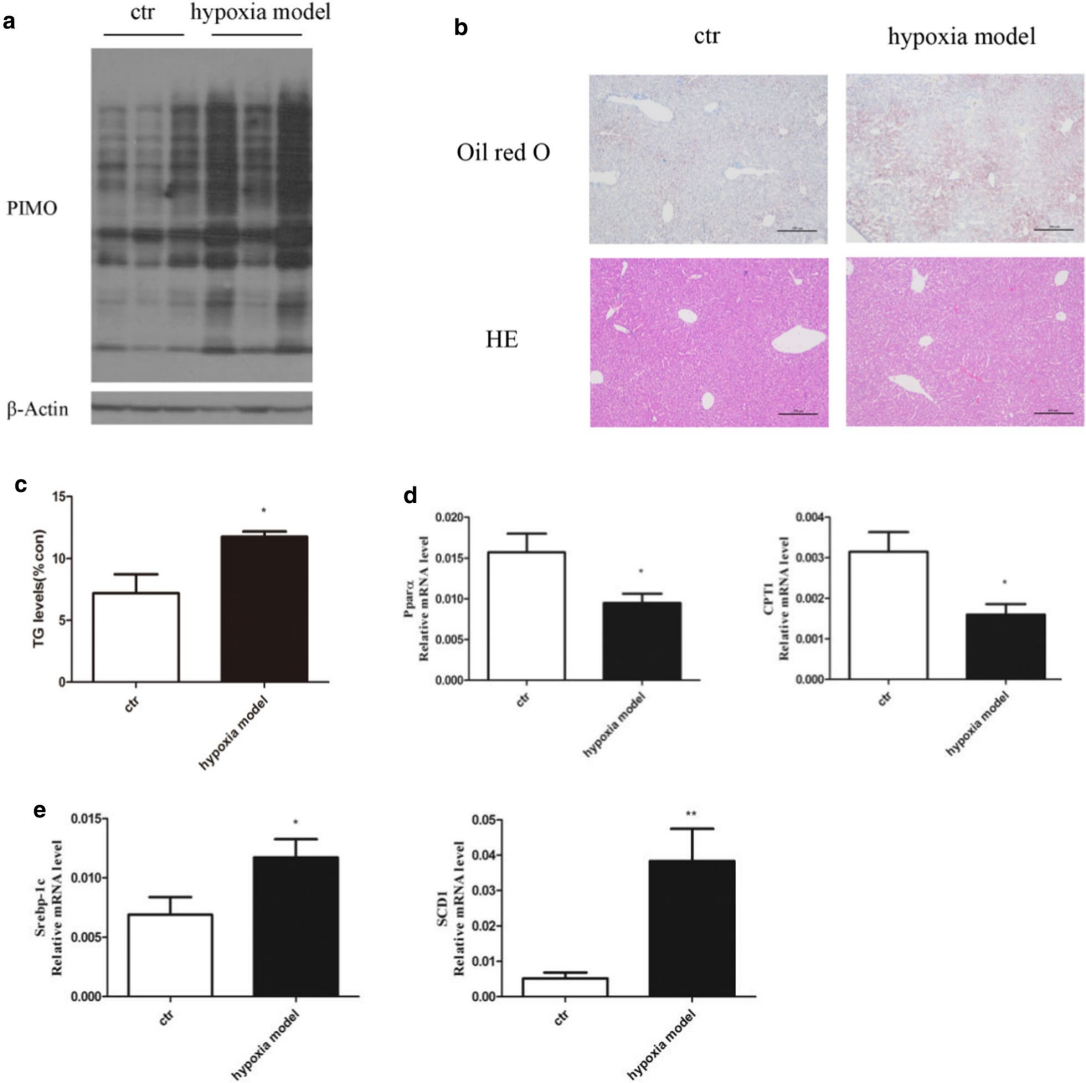
The effect of hypoxia on hepatic TG accumulation. **a** Expression of hypoxic proteins; **b** Oil red O and HE staining (original magnifications, × 100); **c** TG levels; **d** Expression of lipogenic and lipolysis genes. Data are shown as the mean ± SEM, n = 4–6. *P < 0.05; **P < 0.01

We observed robust accumulation of hypoxic proteins in the liver of mice housed in a hypoxic environment, indicating the occurrence of hepatic hypoxia (Fig. 4a). Concomitantly, we also observed hepatic steatosis at pericentral region of the liver acinus via Oil-Red O staining and measurement of live TG content (Fig. 4b, c). To explore the mechanism of this aberrant lipid accumulation in the liver, we measured the gene expression of two pairs of key factors regulating lipid metabolism, the lipogenesis transcription factor SREBP-1c and its downstream target SCD-1, and the lipolysis transcription factor PPARα and its downstream target CPT-1. We found that PPARα and CPT-1 were significantly downregulated, while SREBP-1c and SCD-1 were upregulated in mice housed in the hypoxic chamber (Fig. 4d, e).

## Discussion

NAFLD is a common liver disease with increasing incidence worldwide, and its prevalence is usually parallel to the epidemic of type-2 diabetes and obesity [15]. Due to the insufficient understanding of its pathogenic mechanisms, NAFLD lacks sufficient therapeutic options except life-style intervention including diet and exercise. Thus illustration of NAFLD pathogenic mechanisms will largely facilitate the development of new therapeutic options. Here we showed that high-fat diet induced hepatic steatosis, and concomitantly provoked hepatic hypoxia in mice. Mice housed in the hypoxic chamber developed hepatic-hypoxia as well as steatosis. Thus high-fat diet (HFD) and HFD-induced hypoxia might act as reciprocal causation in the development and/or progression of NAFLD.

OSA, characterized by intermittent hypoxia, is closely linked with NAFLD, implying that intermittent hypoxia (IH) may induce NAFLD or predispose the patients to NAFLD [16, 17]. Researchers did observe that IH exposure increased fasting serum triglycerides (TG) levels as well as hepatic TG content in mice [18]. Hypoxia exerts systemic effects in a variety of diseases, including malignant tumors, rheumatoid arthritis, atherosclerosis, wound healing and bacterial infection. But the relationship between hypoxia and NAFLD is still not fully clarified. Here we investigated the effects of HFD and HFD-provoked hypoxia in the pathogenesis of NAFLD, using a mouse model of NAFLD induced by HFD feeding. We confirmed that HFD feeding resulted in a profound TG deposition in hepatocytes (Fig. 1c, d), and provoked severe hepatic hypoxia as well (Fig. 1e). Interestingly, when we conducted the experiments in cultured primary hepatocytes, we found that the saturate free fatty acid (FFA)- palmitate provoked marked hypoxia 2 h after FFA administration (Fig. 2b, c), while the appearance of fat accumulation peaked at 24 h (Fig. 2a). Consistently, a robust activation of HIF-1α, and HIF-2α to a lesser extent was observed in palmitate acid treated primary hepatocytes (Fig. 2d), confirming that a hypoxic situation was provoked. Furthermore, when hepatocytes were treated with palmitate acid, the activation of HIF and the appearance of hypoxia precede TG accumulation, implying that hypoxia provoked by FFA alone might exert lipogenic effects.

Indeed, we confirmed that hypoxia significantly promoted TG accumulation in vivo in mice housed in a hypoxic chamber (Fig. 4b, c) and in vitro in 0.4 mM CoCl_2_ or 1% oxygen treated primary hepatocytes (Fig. 3a). Consistently, the gene expression of lipolytic transcription factor PPARα and its downstream target CPT-1 was significantly down-reglulated in hypoxia-treated mice (Fig. 4d), while the gene expression of lipogenic transcription factor and its downstream target SCD-1 was upregulated (Fig. 4e). Thus we showed here that HFD and FFA treatment resulted in TG accumulation as well as hypoxia in mice and in cultured hepatocytes respectively, and hypoxia tended to happen earlier and to a deeper extent. Conversely, hypoxia treatment induced TG accumulation in mice and in cultured cells.

Hypoxia-inducible factors (HIFs) are ancient transcription factors that are stabilized when the availability of oxygen is limited (that is, during hypoxia). HIFs could drive a transcriptional program promoting hypoxia adaptation [19]. In HFD-fed mice, HIF-1α mRNA level was significantly upregulated, and direct activation of HIF-2α induced hepatic lipid accumulation, inflammation and fibrosis [10, 11]. In this study, we did observe that HIF-1α and HIF-2α were significantly activated by palmitate acid and CoCl_2_ treatment. Considering the important role of HIFs in the pathogenesis of NAFLD, we speculated that HIF should play a predominant role in FFA- and hypoxia-induced hepatic steatosis. Unexpectively, when we eliminated HIF-1α and HIF-2α action by siRNA in cultured hepatocytes, we did not observe any alteration of TG accumulation in cells with or without HIFs, suggesting that FFA and CoCl_2_ induced hepatic steatosis is not mediated by HIF, at least in cultured hepatocytes. Similar to our observations, using a mouse model with specific hepatic-HIF-1α deficiency, Mesarwi et al. revealed that although HIF-1α was required for the pathogenesis of liver fibrosis, while it did not have a role in the hepatic TG accumulation [19], and it might even play a protective role in non-alcoholic fatty liver disease by reducing lipotoxicity [20].

## Conclusions

In summary, we found that HFD induced hepatic steatosis and concomitantly provoked hepatic hypoxia, which might then act as both inducer and effector in the pathogenesis and/or progression of NAFLD via a HIF-independent pathway. Amelioration of systemic or hepatic hypoxia, instead of blockade of HIF, should be considered for NAFLD therapy, especially in those with OSA.

## Data Availability

The datasets used and/or analysed during the current study available from the corresponding author on reasonable request.

## Abbreviations

NAFLD: Non-alcoholic fatty liver disease
HIF: Hypoxia inducible factor
NASH: Nonalcoholic steatohepatitis
OSAS: Obstructive sleep apnea syndrome
OSA: Obstructive sleep apnea
IH: Intermittent hypoxia
TG: Triglyceride
FFA: Free fatty acid
SCD1: Stearoyl-CoA desaturase 1
CPT1: Carnitine palmitoyl transferase1
PPARα: Peroxisome proliferator-activated receptor alpha
SREBP-1c: Sterol regulatory element-binding protein 1c
